# Hedgehog Signaling in Organogenesis and the Tumor Microenvironment

**DOI:** 10.3390/ijms21082788

**Published:** 2020-04-17

**Authors:** Tsuyoshi Shimo

**Affiliations:** Division of Reconstructive Surgery for Oral and Maxillofacial Region, Department of Human Biology and Pathophysiology, School of Dentistry, Health Sciences University of Hokkaido, Hokkaido 061-0293, Japan; shimotsu@hoku-iryo-u.ac.jp; Tel./Fax: +81-133-23-1429

The Hedgehog signaling pathway was first discovered in 1980 during a large-scale genetic screening seeking to find mutations that affect larval body segment development in the fruit fly, *Drosophila melanogaster* [1]. The Hedgehog signaling pathway is an evolutionarily conserved pathway that governs complex developmental processes including stem cell maintenance, proliferation, differentiation, and patterning. Several recent studies have shown that the aberrant activation of Hedgehog signaling is associated with neoplastic transformation, cancer cell proliferation, metastasis, multiple cancers’ drug resistance, and survival rates. This Special Issue focuses on several aspects of Hedgehog signaling in organogenesis and the tumor microenvironment, and we called for reviews and original papers on the recent efforts in the field of Hedgehog signaling.

This Special Issue of the *International Journal of Molecular Sciences*, entitled “Hedgehog Signaling in Organogenesis and the Tumor microenvironment”, thus includes four original articles and five reviews that provide new insights regarding the roles of Hedgehog signaling in organogenesis and the tumor microenvironment.

Tarulli et al., report on “Discrete Hedgehog Factor Expression and Action in the Developing Phallus”, and they describe a potential developmental interaction involved in urethral closure that mimics bone differentiation and incorporates discrete Hedgehog activity within the developing phallus and phallic urethra [2].

Takebe et al., examined Gli-CreERT2; tdTomato mice, and they demonstrate that the SHH-Gli1 signaling pathway is involved in intramembranous and endochondral ossification during the fracture healing process [3].

Takabatake et al., describe “The Role of Sonic Hedgehog Signaling in the Tumor Microenvironment of Oral Squamous Cell Carcinoma”, and their findings revealed that (1) autocrine effects of SHH induce cancer invasion and (2) paracrine effects of SHH govern parenchyma–stromal interactions of oral squamous cell carcinoma [4].

El Shahawy et al., propose that “Sonic Hedgehog Signaling Is Required for Cyp26 Expression during Embryonic Development”, and they explain that rigidly calibrated Hedgehog and retinoic acid activities are required for normal organogenesis and tissue patterning [5].

Hosoya et al., provide an overview of recent advances related to the role of SHH signaling in tooth development, homeostasis, regeneration, and the regulatory mechanism of stem cell properties in the dental mesenchyme from experiments using tamoxifen administration in iGli1/Tomato mice [6].

Jeng et al., extensively review the recent progress made in the field of “Sonic Hedgehog Signaling in Organogenesis, Tumors, and Tumor Microenvironments”, focusing on the combined use of SHH signaling inhibitors and chemotherapy/radiation therapy/immunotherapy targeting cancer stem cells [7].

Hyuga et al., contribute a comprehensive overview of the “Hedgehog Signaling for Urogenital Organogenesis and Prostate Cancer: An Implication for the Epithelial-Mesenchyme Interaction (EMI)” and compare possible similarities and divergences in Hedgehog signaling functions and the interaction of this signaling with other local growth factors between organogenesis and tumorigenesis. They discuss two pertinent research aspects of Hedgehog signaling: (1) the potential signaling crosstalk between Hedgehog and androgen signaling and (2) the effect of Hedgehog signaling between the epithelia and the mesenchyme on the status of the basement membrane with extracellular matrix structures located on the epithelial–mesenchymal interface [8].

Bechtold et al., offer a thorough review of the recent progress made in studies on the roles of Indian Hedgehog signaling in temporomandibular joint (TMJ) formation, and they discuss important findings regarding the involvement of Hedgehog signaling in TMJ development during embryonic and early postnatal stages as well as in the establishment and postnatal maintenance of TMJs, plus the possible involvement of Hedgehog pathways in osteoarthritic conditions [9].

Haraguchi et al., provide a detailed discussion about “Recent Insights into Long Bone Development: Central Role of Hedgehog Signaling Pathway in Regulating Growth Plate”, and they review the multiple roles of the Hedgehog pathway in the regulation of growth plate formation and differentiation, as well as longitudinal bone development and skeletal disorders [10].

The Editor hopes that these articles will help readers update their knowledge about the role of Hedgehog signaling in physiology and pathology. The efforts of the authors who contributed their excellent articles to this Special Issue are greatly appreciated.
